# Exploring the Impact of Electronic Medical Record–Enabled Versus Paper-Based Systems on the Quality of Nursing Handover: Comparative Case-Study

**DOI:** 10.2196/85909

**Published:** 2026-05-12

**Authors:** Lisa Browning, Urooj Raza Khan, Sandra Leggat, Nicholas Monypenny, James H Boyd

**Affiliations:** 1 Department of Public Health, La Trobe University, CS 1 Building, 1 Kingsbury Drive, Bundoora, 3086, Australia, 61 1300 135 045; 2 Nursing and Midwifery Professional Reports, Office of the Chief Nursing and Midwifery Officer, Eastern Health, Box Hill, Australia; 3 Australasian Institute of Digital Health, South Melbourne, Australia; 4 Eastern Health Institute, Eastern Health, Box Hill, Australia; 5 E-Health Program, Infrastructure and Digital, Eastern Health, Box Hill, Australia

**Keywords:** nursing handover, electronic medical record, electronic health record, health information systems, patient engagement

## Abstract

**Background:**

Ineffective clinical handover has the potential to compromise patient safety and quality of care. Standardizing the handover process is a widely adopted improvement strategy intended to reduce failures of information transfer. By enabling real-time access to patient information, electronic medical records (EMRs) could address communication issues inherent to nursing handover.

**Objective:**

This case study sought to compare the quality of nursing handover occurring at EMR-enabled sites with that occurring at paper-based sites, within a single Australian public health service.

**Methods:**

A comparative case study design was used, using quantitative data collected from observational audits of 60 handovers and posthandover surveys conducted in EMR-enabled and paper-based ward environments. Handover quality was measured through compliance with the organization’s Clinical Handover Standard and staff-reported perceptions, enabling comparison between cohorts.

**Results:**

Compared with paper-based wards, EMR-enabled wards demonstrated more consistent communication of clinical alerts and risks and fewer interruptions, whereas paper-based wards showed higher rates of bedside handover and patient engagement.

**Conclusions:**

EMR implementation alone does not ensure high-quality nursing handover. EMR interface design and functionality may act as a barrier to bedside handover and patient engagement, and contribute to continued reliance on paper-based artifacts. Targeted EMR design and nursing informatics-led optimization are required to better support nursing handover as a complex and cognitively challenging communication process.

## Introduction

### Overview

Nurses play a vital role in determining the clinical outcomes of their patients, particularly in relation to ensuring patient safety and continuity of care [1]. Extensive research demonstrates that deficiencies in clinical communication processes represent a significant risk to patient safety [2] and are a common source of sentinel events [3]. When communication is incomplete, unclear, or inaccurate, patient safety may be compromised [1].

Clinical handover is a critical and complex health care communication process, serving as the primary mechanism for transferring responsibility and accountability between shifts [4]. Effective and high-quality nursing handover is a core patient safety mechanism, facilitating the timely, accurate, and efficient exchange of clinical information [5]. Consistent with the National Safety and Quality Health Service (NSQHS) Standards, effective handover requires information to be clear, comprehensive, and precise to support safe, appropriate, and continuous care [6].

Conversely, inadequate or poorly executed clinical handovers can adversely affect patient outcomes [5]. Ineffective communication during handover has been associated with inaccurate clinical assessments [7], delayed diagnoses [6], inappropriate treatments, and medication errors [5]. The impacts of these failures range from minor disruptions to care to severe and potentially catastrophic outcomes. As health care systems increasingly adopt electronic medical records (EMRs) to support clinical communication, understanding how these systems influence the quality and safety of nursing handover is essential.

### Background

Efforts to structure and standardize nursing handover processes are often recognized as key improvement strategies for improving the quality and safety of clinical communication [8]. Standardized communication protocols and structured handover tools such as ISOBAR (Identification Situation Observations Background Assessment/Action Recommendation) [9] are “intended to prevent failures of information transfer” [10] by ensuring that information shared is relevant, concise, and focused [5]. Handover mnemonics like ISOBAR reduce the cognitive workload associated with performing handover [11,12] and were designed to overcome differences in communication styles, a factor contributing to communication errors [13]. By streamlining the handover process, these tools also enhance situational awareness within team-based environments, supporting the development of a shared mental model of a patient’s clinical status [13,14]. Standardizing handover processes is therefore a frequent recommendation aimed at improving handover quality and patient outcomes.

In Australia, NSQHS Standards provide a quality assurance mechanism and “a nationally consistent statement about the standard of care consumers can expect from their health service organizations” [15]. NSQHS Standard 6, “Communicating for Safety includes the criterion for communication at clinical handover,” encompassing structured clinical handover, defining minimum information content of handover, key principles of clinical handover, and engaging patients and caregivers in clinical handover processes [15].

Increasingly, bedside handover has been adopted as standard practice in Australian hospitals [1] and is promoted as a strategy to enhance patient engagement, transparency, and patient-centered care [16]. Best practice bedside handover requires nurses to communicate accurate, comprehensive, and contemporaneous clinical information in real time [5], often in the presence of the patient, their families, and caregivers. While this model of handover offers important benefits [1,16], it also increases the complexity of information exchange [17], requiring nurses to synthesize and communicate large volumes of clinical data efficiently [18] while maintaining patient engagement [16] and situational awareness [11,13]. These demands place heightened emphasis on the availability, accuracy, and usability of clinical information at the point of care, and on the systems through which that information is accessed and communicated during handover.

In this context, EMRs have been introduced internationally as a means of supporting complex clinical communication processes, including nursing handover. EMRs are clinical information systems that facilitate real-time access to patient data and provide structured system-level processes intended to support patient safety, quality improvement, and compliance with expected standards of practice [19,20]. Key patient safety mechanisms include electronic prescribing and medication administration, the use of alerts, checklists, and predictive tools and embedded evidence-based practices, supporting decision making at the point of care [19,21]. Beyond strengthening compliance, EMRs also enable continuous quality improvement by generating routinely collected data that can be used to monitor performance, identify variations in practice, and inform iterative improvement cycles [19].

With real-time access to up-to-date patient information, EMRs have been proposed as a means to address the communication challenges inherent to clinical nursing handover [9]. EMRs have the potential to support nursing handover through the use of structured EMR-based handover tools, thus aligning with the intent of NSQHS Standard 6, by supporting timely, accurate, and standardized communication of critical patient information [5].

The introduction of an EMR in itself does not guarantee improvements in clinical communication or patient safety. The literature remains equivocal regarding the extent to which EMRs improve efficiencies in health care, with some studies noting potential adverse effects on nursing handover, including increased work and decreased efficiencies [22,23]. Poorly planned or ineffective implementations of an EMR may disrupt established nursing workflows and may fail to provide the required cognitive support nurses require during handover [22,24]. Information fragmentation within EMRs and the need to navigate multiple screens or data sources may further increase nurses’ cognitive workload during handover, introducing new risks for both patients and clinicians [25,26]. Understanding both the intended benefits and unintended consequences of EMR adoption is essential to assessing their value in practice [27,28].

While EMRs provide the technological infrastructure to support structured and standardized handover, the effective translation of EMR functionality into safe and high-quality nursing practice is underpinned by nursing informatics capability. Nursing informatics describes the knowledge and skills required by registered nurses to integrate nursing science with information and computer sciences to manage, communicate, and apply clinical data, information, and knowledge in practice [29].

The presence of digital technology alone is insufficient to improve clinical outcomes. Contemporary nursing practice requires nurses to navigate an increasingly complex digital environment, meaningfully engage with digital systems, adapt workflows, and effectively use information within the realities of clinical practice [30,31], including during time-critical activities such as nursing handover. Nursing Informatics, therefore, provides an interpretive and operational lens through which the impact of EMR-facilitated handover can be understood. Within this context, nurses play a critical role in shaping documentation practices, information use, and workflow integration [32,33] in ways that either support or undermine the safety and quality objectives of a structured, best-practice nursing handover, occurring at the patient’s bedside.

While handover standardization alone has the potential to improve the effectiveness and efficiency of nursing handover and contribute to safer patient care [15], high-quality research linking the implementation of EMR-facilitated structured nursing handover to impacts on patient-related outcomes is still lacking. A knowledge gap therefore exists, as does an opportunity for further exploration of EMR-mediated solutions that may serve to support, standardize, and therefore enhance nursing handover.

Despite the widespread implementation of EMRs and increasing adoption of bedside handover, there remains limited empirical evidence examining how EMR-facilitated structured nursing handover translates into practice, particularly when compared with handover occurring in non-EMR environments within the same health service. Understanding how differences in information systems, workflow integration, and nursing informatics capability influence the quality of handover is essential to determining whether EMRs achieve their intended safety and quality objectives.

This gap is particularly salient for health services implementing EMRs in a staged or hybrid manner, where variations in digital maturity create a natural opportunity to examine the impact of EMR-enabled handover in context. It is within this organizational and digital landscape that the present case study was situated.

### Context

Eastern Health is a large public health service situated in Victoria, Australia, serving a wide catchment across Melbourne’s east. The organization spans 6 local government areas, covering 2816 square kilometers and comprising 21 health service locations. Eastern Health’s 3 largest acute hospital sites are Box Hill Hospital, Maroondah Hospital, and Angliss Hospital.

A full EMR was implemented at Box Hill Hospital in October 2017. At Angliss Hospital, Electronic Medication Prescribing and Administration was implemented in November 2016; however, inpatient clinical documentation has remained predominantly paper-based. At the time of the study (conducted March to May 2023), implementation of the full EMR across inpatient wards at Maroondah Hospital (including Electronic Medication Prescribing and Administration) and completion of the full EMR deployment at Angliss were planned for late 2025 and early 2026, respectively. As a result, Eastern Health operated within a hybrid digital environment with varying levels of maturity across sites.

The introduction of full EMR at Box Hill Hospital fundamentally altered the interface through which nurses accessed, synthesized, and communicated clinical information during handover. In contrast, nurses at Maroondah and Angliss continued to rely primarily on paper-based documentation supported by other limited digital systems. These contrasting documentation environments, operating within the same organization, provided a structured basis for systematic comparison of EMR-enabled and predominantly paper-based nursing handover within a shared organizational context.

The sampling frame comprised nursing handovers conducted on all acute general medical and acute general surgical wards across Box Hill, Maroondah, and Angliss Hospitals. Each site has a 24-hour emergency department and intensive care unit, providing comparable patient cohorts within their respective acute medical and acute surgical wards. The selection of these was intended to promote homogeneity between the EMR-enabled and non-EMR sites, thereby minimizing the impact of variability in patient acuity on nursing expertise and handover practices. A total of 12 wards were identified as suitable for inclusion in the study. The target population comprised clinical nursing staff responsible for conducting routine shift-to-shift handovers.

### Study Purpose

The purpose of this case study was to examine and compare the quality of nursing handover conducted in fully EMR-enabled wards at Box Hill Hospital with handover occurring in predominantly paper-based documentation environments at Maroondah and Angliss Hospitals. Handover quality was examined within routine clinical practice and assessed using two complementary measures: (1) the degree of compliance with the organization’s Clinical Handover Standard, and (2) clinical nursing staff’s self-reported perceptions of handover quality.

This purpose aligns with the study’s focus on understanding how differences in documentation environments and information access may shape the conduct and perceived quality of bedside nursing handover within a shared organizational context.

### Aims

The primary aim of this study was to compare the quality of nursing handover in fully EMR-enabled wards and predominantly paper-based wards within acute inpatient settings.

Specifically, the study sought to compare nursing handover practices in fully EMR-enabled wards with those occurring in predominantly paper-based environments, by examining whether EMR presence was associated with:

- Higher-quality nursing handover, as measured by compliance with the organization’s Clinical Handover Standard (Multimedia Appendix 1).
- Differences in duration of nursing handover.
- Differences in the level of patient engagement in the handover process.
- Differences in staff-reported perceptions of handover quality.

## Methods

### Design

To address the study purpose and aims, nursing handover practices were examined across EMR-enabled and predominantly paper-based wards within the same health service. This study used a comparative quantitative case-study design using 2 complementary quantitative data sources.

Case study research is “a distinctive form of empirical enquiry” [34] that investigates a contemporary phenomenon within its real-life context [35]. This approach is well-suited to health care settings, where complex clinical processes can be examined using observational methods alongside other data sources, allowing meaningful characteristics of real-world practice to be retained [34], while generating opportunities for more comprehensive explanations [36].

As described within the context section of this case study, the staged implementation of the EMR across Eastern Health provided a naturalistic opportunity to apply a comparative case study design, enabling examination of nursing handover practices in wards supported by a fully implemented EMR alongside those operating in predominantly paper-based environments. Comparative evaluations of health information systems remain relatively uncommon, with much of the existing literature relying on descriptive or correlational approaches [27]. As such, this design offered the potential to generate contextually grounded insights relevant to both local system optimization and the broader evidence base on formative evaluations of digital health systems.

This case study used 2 quantitative data collection instruments. First, structured observational audits of routine shift-to-shift nursing handover were conducted to assess handover quality based on observed compliance with key principles of Eastern Health’s Clinical Handover Standard. Second, posthandover surveys were administered to capture clinical nursing staff’s perceptions of the quality of handover received, with responses quantified using Likert-scales, responses to closed yes/no questions, and counts of affirmative responses. For both data sources, comparisons were undertaken between handover occurring in the EMR-enabled wards and those in predominantly paper-based wards. This multi-source quantitative approach enabled direct observation of the phenomenon being studied alongside quantified staff perceptions of handover quality [34].

Consistent with guidance on health information system evaluation, the use of multiple data sources allowed complementary insights to be integrated, supporting a more comprehensive understanding of nursing handover within differing documentation environments [27]. EMR-supported handover was examined from a nursing informatics perspective, focusing on nurses’ use of available documentation systems and the clinical information exchanged during routine handover practice.

The data collected and analyzed in this case study form part of a broader program of research examining nursing handover processes within EMR-enabled clinical environments. Subsequent analyses will explore nurses’ interaction with, and use of, EMR functionality during handover in greater depth.

### Participants

The study was conducted across acute general medical and acute general surgical inpatient wards within 3 hospital sites of a single public health service in Victoria, Australia. Participants in the observational audits and posthandover surveys were nurses conducting routine shift-to-shift nursing handovers on the 12 selected wards during the data collection period. The distribution of wards by site, documentation environment, and the number of handovers audited is presented in Table 1.

**Table 1.** Selected wards by site and number of handovers audited.

Site	Medical wards	Surgical wards	Medical record status	Total number of wards	Number of handovers audited
Box Hill	3	3	Full EMR^a^	6	30
Maroondah	2	2	Paper-based	4	20
Angliss	1	1	Paper-based	2	10
Total	6	6	**—^b^**	12	60

a EMR: electronic medical record.

b Not applicable.

For this comparative case study, the case was defined as the selected wards within the 3 hospitals of a single public health service. The unit of analysis was the individual shift-to-shift nursing handover. Data were collected between March and May 2023, across morning and afternoon shifts, encompassing both paper-based and EMR-enabled documentation environments.

### Sampling

A convenience sampling approach was used, whereby nurses giving handover on the selected wards during scheduled data collection periods were invited to participate. To promote variation in observed practice, handovers were sampled sequentially across different days, shifts, and times within each ward to capture variation in routine practice. Five handovers per ward were targeted to ensure adequate representation of routine handover practices within each clinical context, while remaining feasible given the resource-intensive nature of direct observational auditing across multiple sites. Five nursing handovers were sampled per ward, resulting in a total of 60 observed handovers across the 12 wards.

### Inclusion and Exclusion Criteria

Eligibility for participation was defined to ensure consistency across wards and sites.

#### Inclusion Criteria

Participants were eligible if they were registered nurses with current nursing registration, used by Eastern Health, and scheduled to participate in shift-to-shift nursing handover during the data collection period. All participants were required to provide informed consent.

#### Exclusion Criteria

Undergraduate nurses, assistants in nursing, agency nurses, and staff who declined to participate or did not consent to the collection of basic demographic information were excluded from the study.

### Ethical Considerations

Ethics approval was obtained from Eastern Health’s Human Research Ethics Committee, HREC number LR22-042-84698. Reciprocal ethics approval was obtained from La Trobe University. Written informed consent was obtained from all nurse participants involved in the observational audits and posthandover surveys, including nurses giving and receiving handovers. Patients whose handovers were observed were provided with written information about the observational audit in advance. Patients were informed that no personal identifiers or health information would be collected or recorded as part of the audit. Verbal consent to proceed with handover observation was obtained from patients immediately prior to handover commencement. Participants received no financial or other compensation for their participation.

### Data Collection

#### Overview

A total of 60 observational audits and corresponding posthandover surveys were conducted between March and May 2023. Observational audits occurred during the morning-to-afternoon (AM to PM) shift-to-shift nursing handover. All observational audits were undertaken by a single auditor to ensure consistency. Both the observational audit tool and the posthandover survey were completed using paper-based instruments at the point of data collection. Immediately following each observed handover, the nurse receiving the handover completed a posthandover survey. All paper-based data were subsequently entered into REDCap by the same auditor later on the day of collection, following completion of data collection activities for that day.

Each nurse was observed delivering handover once; however, nurses may have received handover and completed the posthandover survey on more than one occasion, consistent with the sampling approach and routine practice, resulting in up to 120 participants.

For each participating ward, 5 observational audits and posthandover surveys were completed over a period of 1 to 2 days. The observational audits comprised 30 handovers in fully EMR-enabled wards and 30 handovers conducted and observed in predominantly paper-based documentation environments. As this study represented an observational comparative case study and an exploratory examination of nursing handover practices, formal sample size or power calculations were not undertaken.

#### Observational Audit

The observational audit was designed to quantify the quality of nursing handover by measuring compliance with the organization’s Clinical Handover Standard (Multimedia Appendix 1). This Standard addresses 11 key principles underpinning best practice clinical handover and is aligned with the NSQHS Standard 6: Communicating for Safety. These principles are summarized in Table 2.

**Table 2.** Principles for best practice handover. Extracted from the Eastern Health Clinical Handover Standard, November 2022, available in Multimedia Appendix 1.

Number	Principle
1	Are undertaken using a consistent and structured process, “ISOBAR,”^a^ which includes a minimum dataset.
2	Recognize that clinical handover requires effective communication between clinicians, including an opportunity to clarify information.
3	Maintain the patient’s right to confidentiality and privacy; sharing of information is based upon the relevance and impact to care and outcomes
4	Promote inclusion of patients and, where appropriate, their caregivers, while acknowledging that sensitivity is required where other patients or visitors may overhear patient information.
5	Consider the need to engage interpreters if patients have communication difficulties, such as cultural and linguistic diverse backgrounds or hearing impairments.
6	Occur prior to, or at the time when clinician/s transfer care and accountability, and acknowledge the transfer of accountability for some or all of the patient’s care.
7	Ensure adequate preparation prior to undertaking a clinical handover, making certain that the process is efficient and that all relevant information is transferred.
8	The clinician providing direct care leads the clinical handover, and where possible, handover occurs as face-to-face communication.
9	Respect the importance of handover, with minimal interruptions or distractions.
10	Within mental health settings, a team handover occurs prior to individual handover to ensure all staff are aware of the current milieu and risk issues present within the unit.
11	Documentation of the handover is recorded within the progress notes, or equivalent, in accordance with the Clinical Documentation Standard.

a ISOBAR: Identification Situation Observations Background Assessment/Action Recommendation.

Health service organizations are required to implement governance frameworks, systems, and processes that support effective clinical communication during handover. Locally contextualized clinical handover standards provide a mechanism through which NSQHS requirements are operationalized at the organizational level, thereby supporting patient safety and quality of care [15].

#### Audit Tool Development

The observational audit tool was developed to assess whether handovers complied with established principles of effective clinical communication. Tool development occurred in partnership with Eastern Health’s Communicating for Safety Clinical Risk Governance Committee (CFS CRGC) to ensure content validity and alignment with established clinical governance standards. Audit questions were aligned with those used in previous organization-wide Clinical Nursing and Midwifery Handover Audits, enabling comparison with existing organizational data and reinforcing alignment with established quality and safety processes.

In determining the final audit tool, several decisions were made:

- Two principles of the Clinical Handover Standard (Principles 10 and 11) were considered outside of the scope of this study and were therefore excluded.
- The location of handover was identified as a key variable of interest in the context of a newly introduced digital interface. Specified within the Standard is that “where possible, clinical handover will occur face to face and in the patient’s presence (bedside handover).” This element was therefore included as a distinct principle for auditing.
- Handover efficiency was measured through handover duration and whether handover commenced and concluded on time, with all required staff present.
- Respect for the handover process was quantified by recording the number of interruptions occurring during each handover episode.

Ten principles were therefore included in the final audit framework. A total of 28 audit questions were used to assess compliance with these principles. Each question required a yes or no response, with the exception of interruptions and handover duration, which were recorded as counts and time (minutes), respectively. The full list of audit questions and their alignment with each principle is presented in Table 3.

**Table 3.** Observational audit questions in relation to each principle.

Principle	Questions	Response	Interpretation and data analysis
Handover occurs at the bedside	Did handover occur at the bedside?	Y/N	% compliance with the principle per ward/cohort/overall.
Handover follows ISOBAR^a^	Identify: Was the person/team involved in the handover introduced to the patient? Identify: Was a 3-point ID check completed? Situation: Was the current clinical situation of the patient (ie, stable, improving, deteriorating, and discharge plan) discussed? Observation: Were recent clinical observations discussed? Background: Was the relevant patient background/history, evaluation, and management to date shared? Assessment: Were relevant clinical assessments, any concerns from assessments, or outstanding assessments/actions discussed? Assessment: Were clinical risks (ie, falls, pressure injuries, delirium, malnutrition, behavior of concern [BOC]) discussed? Assessment: Were clinical alerts (ie, safety concerns such as aggression/harms to others, infection control) discussed? Request/recommendation: Were actions required and recommendations for ongoing care discussed, including responsibility for actions and agreed transfer of responsibility?	Y/N	% compliance for each question and % compliance with the principle per ward/cohort/overall.
Communication is effective	Did the nurse leading the handover provide an opportunity to ask questions? Did the nurse receiving the handover ask any questions to clarify the information received? Did the nurse receiving the handover ask any questions to seek information that was not offered?	Y/N	Principle deemed met if any 1 question answered in the affirmative and % compliance with the principle per ward/cohort/overall.
Patient’s right to confidentiality is maintained	Was all the information provided at the bedside clinically relevant, avoiding information pertaining to sensitive social or clinical conditions?	Y/N	—^b^
Inclusion of the patient, family, or caregiver is promoted	Was the patient greeted during the handover process? Was the handover process explained to the patient? Was the patient and caregiver/family member present included in the handover? Was the patient given an opportunity to ask questions or discuss goals or raise concerns?	Y/N	% compliance for each question and % compliance with the principle per ward/cohort/overall.
Interpreters are used where needed	Is the patient’s first language or native language a language other than English? Is there a language barrier present that would prevent the patient from being included in the handover? Was an interpreter used during handover? Was the future planning or booking, or did an interpreter discuss?	Y/N	% compliance with the principle per ward/cohort/overall. Logic used: If N recorded for Q1 or Q2=N/A If Y recorded for Q1 & Q2 (interpreter deemed needed) AND Y recorded for either Q3 OR Q4 (interpreter is used or planned)=Y/N.
Transfer of care and accountability is acknowledged	Was there an acknowledgment of a transfer of care or accountability? *SELECT ALL THAT APPLY: Discussion of actions required and responsibility for actions occurred Review of treatment and care plan, clinical risks, management, and recommendations occurred Review of information and clarification sought by the recipient Acknowledgment and agreement from the recipient of the plan of care	Y/N. Principle deemed met if any 1 question answered in the affirmative	% compliance with the principle per ward/cohort/overall.
The direct care clinician leads handover and, where possible, delivered face to face	Did the clinician providing direct care lead the handover? Was the handover delivered face to face?	Y/N	% compliance for each question and % compliance with the principle per ward/cohort/overall.
Respect for process – minimized interruptions and distractions	Was the handover interrupted?	Number of interruptions recorded	Number of interruptions recorded per ward/cohort/overall.
Handover is efficient	Time taken to deliver the handover	Duration of handover in minutes (rounded)	Duration of handover per ward/cohort/overall.
Handover is efficient	Handover started and finished on time, with all required staff present?	Y/N	% compliance for the question.

a ISOBAR: Identification Situation Observations Background Assessment/Action Recommendation.

b Not applicable.

#### Posthandover Survey

The posthandover survey was designed to complement the objective data captured in the observational audits by capturing the immediate assessment of handover quality from the perspective of the handover recipient. Survey items were developed to quantify staff perceptions of satisfaction, quality, efficacy, and efficiency associated with each handover episode. This approach is consistent with the literature, which emphasizes the importance of accurate and comprehensive communication during clinical handover to support patient safety [6].

The survey was developed collaboratively with the organization’s CFS CRGC to ensure alignment with clinical handover principles and organizational standards, and comprised 11 items: 5 Likert-scale questions, 5 closed-ended (yes/no) questions, and 1 free text response. Likert-scale items were scored on a 5-point scale, with a composite score (out of 25) calculated for questions assessing satisfaction, quality, and perceived efficacy. Closed-ended items were analyzed using frequency counts and percentages. Free-text responses were reviewed descriptively; however, due to limited volume and specificity, they were not subjected to formal qualitative analysis. The full list of posthandover survey questions, response formats, and corresponding analytical approaches is provided in Table 4.

While formal psychometric analyses (eg, internal consistency) were not feasible due to the exploratory nature of the study, sample size, methodological triangulation with the observational audits supported interpretive validity.

**Table 4.** Posthandover survey questions.

Question	Construct	Response	Interpretation and data analysis
Are you satisfied with the handover you just received?	Satisfaction	Likert scale 1‐5: 1=very dissatisfied 2=dissatisfied 3=neither satisfied nor dissatisfied 4=satisfied 5=very satisfied	Score (out of 5) for each question per ward/cohort/overall Composite score (out of 25) for questions 1‐5 per ward/cohort/overall
Rate the overall quality of the handover received	Quality	Likert scale 1‐5: 1=very poor 2=poor 3=average 4=good 5=excellent	Composite score (out of 25) for questions 1‐5 per ward/cohort/overall
Did the handover follow the ISOBAR format?	Quality	Likert scale 1‐5: 1=strongly disagree 2=disagree 3=neither agree nor disagree 4=agree 5=strongly agree	Composite score (out of 25) for questions 1‐5 per ward/cohort/overall
Rate how accurate you believe the handover was?	Efficacy	Likert scale 1‐5: 1=very poor 2=poor 3=average 4=good 5=excellent	Composite score (out of 25) for questions 1‐5 per ward/cohort/overall
Rate how comprehensive you believe the handover was?	Efficacy	Likert scale 1‐5: 1=very poor 2=poor 3=average 4=good 5=excellent	Composite score (out of 25) for questions 1‐5 per ward/cohort/overall
Was the required content effectively communicated?	Efficacy	Y/N	% occurrence (count of yes) per ward/cohort/overall
Were you given the opportunity to clarify information?	Efficacy	Y/N	% occurrence (count of yes) per ward/cohort/overall
Was there any specific information you felt was missing?	Efficacy	Y/N	% occurrence (count of no) per ward/cohort/overall
If Y to Q8: In a few short words, briefly state what information you believe was missing	Efficacy	Free text	To be themed where possible
Was the duration of handover appropriate?	Efficiency	Y/ N - too short N - too long	% occurrence (count of yes) per ward/cohort/overall
Was the inclusion of the patient, family, or caregiver promoted?	Quality	Y/N	% occurrence (count of yes) per ward/cohort/overall

It should be noted that staff perceptions of patient engagement were higher than what was observed in audits, suggesting possible overestimation due to social desirability bias or limited understanding of effective patient inclusion during handover. This discrepancy highlights the importance of combining objective and self-reported measures when evaluating handover quality.

### Data Analysis

#### Overview

Data were extracted from REDCap into Microsoft Excel by the research team and deidentified prior to analysis. All data handling and analysis complied with institutional governance, ethical, and privacy requirements.

#### Observational Audits

For each audit question, the frequency of affirmative responses was calculated. Compliance with each handover principle was determined by aggregating question-level responses and summarized at the ward level, by cohort (EMR-enabled vs paper-based), and for the overall sample. Descriptive statistics, including mean and median values where appropriate, were generated to enable comparison between cohorts. The detailed analytic approach, itemized by audit question, is presented in Table 3.

Nonparametric tests (Kruskal-Wallis rank-sum test) were used for intergroup comparisons due to nonnormal distribution of data. Handover duration and frequency of interruptions were analyzed descriptively.

#### Posthandover Surveys

Posthandover survey data were analyzed descriptively. Likert-scale responses were summarized using mean and median values at the ward level, by cohort, and overall. For closed-ended (yes/no) questions, frequencies and percentages of affirmative responses were calculated across the same groupings.

A composite score was derived from the 5 Likert-scale items assessing satisfaction, quality, and perceived efficacy to provide an overall measure of staff perceptions of handover quality. Comparative analysis was undertaken to examine alignment between staff-reported perceptions and observed handover quality as measured through compliance with the Clinical Handover Standard.

The posthandover survey also included a single free-text question inviting respondents to identify any information they believed was missing from handover. Responses were limited and lacked sufficient depth for qualitative analysis and were therefore not subjected to formal qualitative analysis or reported in this paper.

Given the exploratory nature of the study and the relatively small sample size, formal effect size estimation was not undertaken, and results were interpreted primarily descriptively.

### Validity and Reliability

#### Overview

The observational audit and posthandover survey instruments were developed in collaboration with the organization’s CFS CRGC, supporting content validity and ensuring alignment with established clinical governance standards. Audit items were mapped directly to the organization’s Clinical Handover Standard and aligned with the NSQHS Standard 6: Communicating for Safety, ensuring construct relevance and consistency with best-practice frameworks.

To enhance the validity of the findings, objective measures of handover quality derived from observational audit compliance with the Clinical Handover Standard were compared with self-reported staff perceptions of the handover quality captured through the posthandover survey, providing methodological triangulation. Use of a single trained auditor and standardized data collection procedures further supported reliability and methodological rigor.

#### Observational Audits

Design of the observational audits was purposefully aligned with previous organization-wide Clinical Nursing and Midwifery Handover Audits. This alignment supported consistency in measurement and enabled comparison with existing audit data, strengthening confidence in the reliability of the observational audit approach.

#### Posthandover Survey

As the posthandover survey captured individual staff perceptions, inherent limitations relating to subjectivity and response bias are acknowledged. While the survey instrument had not been previously administered within the organization, its development was informed by established handover constructs and aligned with the observational audit framework, supporting interpretive validity.

## Results

### Observational Audits

#### Overview

Observational audit data were first analyzed to compare overall compliance with the Clinical Handover Standard between paper-based and EMR-enabled wards. Compliance with individual principles of the Standard was then examined to identify areas of variation between the 2 cohorts.

#### Overall Compliance With the Clinical Handover Standard

##### Overview

Overall compliance with the Clinical Handover Standard was assessed across 10 key principles. Eight of these principles were evaluated as either met or not met. Table 5 presents percentage compliance for each of these 8 principles, along with overall compliance by cohort, expressed using mean and median values. This composite measure, derived from compliance across the audited principles, indicated broadly comparable overall handover quality between EMR-enabled and paper-based wards.

**Table 5.** Overall compliance with the standard.

Principle	Paper-based^a^		EMR^b^-enabled^c^	
	Mean	Median	Mean	Median
Handover occurs at the bedside (%)	83	80	63	60
Handover follows ISOBAR^d^ (%)	80	78	83	78
Communication is effective (%)	100	100	100	100
Patient’s right to confidentiality is maintained (%)	100	100	100	100
Inclusion of the patient, family, or caregiver is promoted (%)	70	68	43	37
Interpreters are employed where needed	—^e^	—	—	—
Transfer of care and accountability is acknowledged (%)	100	100	100	100
The direct care clinician leads handover and, where possible, delivered face to face (%)	100	100	100	100
Overall compliance (first 8 principles) (%)	90	100	84	100
Handover is efficient (handover duration) (minutes)	3.87	4	4.13	3.5

a Respect for process: 7 interruptions.

b EMR: electronic medical record.

c Respect for process: 7 interruptions.

d ISOBAR: Identification Situation Observations Background Assessment/Action Recommendation

e Not applicable. The principle, “Interpreters are employed as needed,” demonstrated insufficient data for comparative analysis, with only 2 instances of interpreter need identified across the sample. This has been reported descriptively and excluded from composite compliance to avoid disproportionate influence on results.

Paper-based wards demonstrated slightly higher mean overall compliance with the Clinical Handover Standard (90%) than EMR-enabled wards (84%), although median compliance was equivalent across cohorts. This pattern suggests broadly comparable aggregate performance, with observed differences driven by variation across specific handover principles, rather than consistent divergence across all domains.

The 2 remaining principles, “Respect for Process” and “Handover is Efficient,” were assessed using alternative metrics, quantified by the number of interruptions that occurred, and the duration of handover, respectively.

##### Interruptions During Handover

Across the 60 handovers observed, a total of 8 interruptions were recorded. Seven interruptions occurred in 2 paper-based wards, while only one interruption was observed in an EMR-enabled ward.

##### Handover Duration

Median handover duration was slightly longer in paper-based wards (4 min) compared with EMR-enabled wards (3.5 min). Initial analysis was considered using ANOVA; however, the data failed tests of normality (Shapiro–Wilk test for normality). Consequently, nonparametric methods, including the Kruskal–Wallis rank-sum test, were used. This analysis indicated no statistically significant differences between the paper-based and EMR-enabled wards, likely influenced by the small sample size.

**Figure 1.**
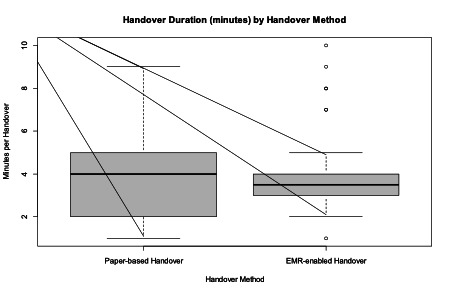
Handover duration by handover method.

As illustrated in Figure 1, the median handover duration was slightly longer for paper-based handover (4 min) compared with EMR-enabled handover (3.5 min), suggesting a marginally quicker handover in EMR settings. However, the IQR was wider for paper-based handovers (2.75 vs 1), indicating greater variability within the middle 50% of values, suggesting more consistent handover duration in EMR-enabled settings.

### Compliance Against Individual Principles of the Standard

#### Overview

Four principles demonstrated 100% compliance across all 12 wards:

- Communication is effective,
- Patient’s right to confidentiality is maintained,
- Transfer of care and accountability occur,
- Direct care clinician leads handover.

Analysis of the remaining principles revealed several areas of divergence between cohorts, as outlined below.

#### Compliance With ISOBAR

**Figure 2.**
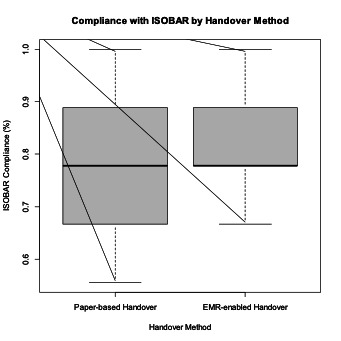
Compliance with ISOBAR by handover method. EMR: electronic medical record; ISOBAR: Identification, Situation, Observations, Background, Assessment/Action, Recommendation.

Compliance with all 9 ISOBAR elements was reviewed for both cohorts. Median compliance scores were equivalent between cohorts (0.778); however, EMR-enabled wards demonstrated a slightly higher mean compliance score (0.833) compared with paper-based wards (0.804). Paper-based wards exhibited greater variability, reflected by a wider IQR (0.194 vs 0.111). Both cohorts achieved the maximum possible compliance score of 1.0. These findings are illustrated in Figure 2.

**Figure 3.**
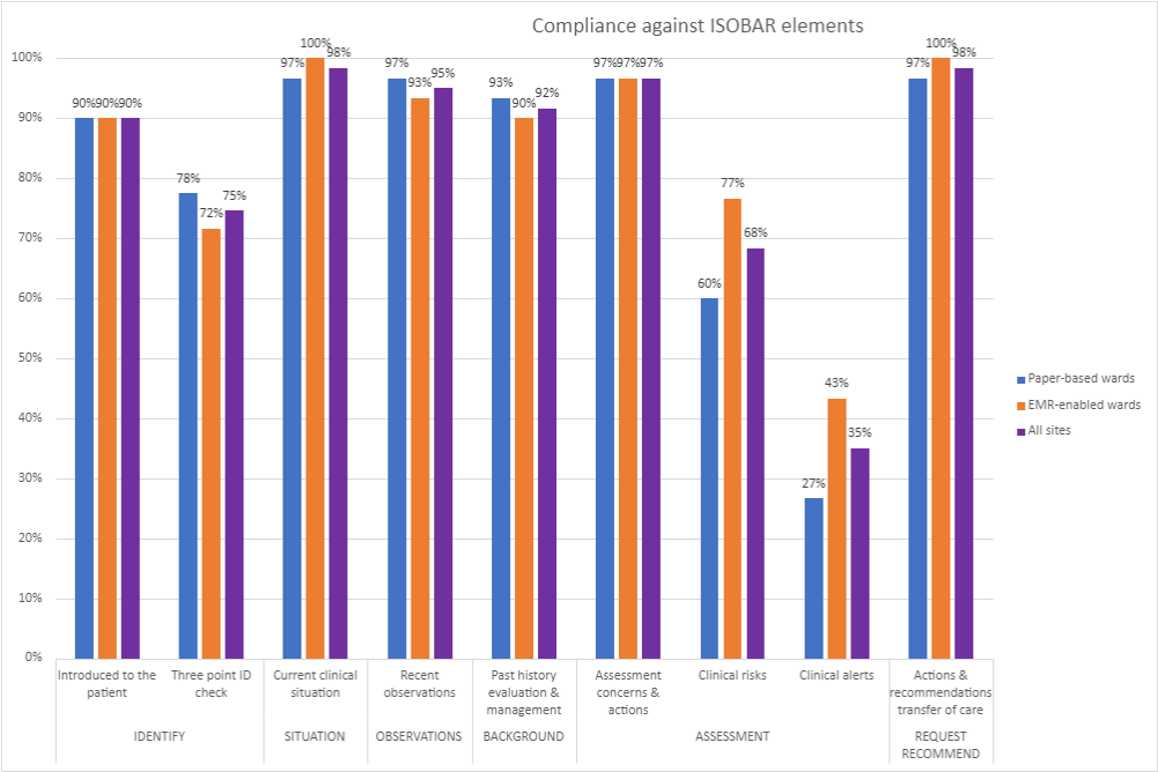
Compliance against ISOBAR elements. EMR: electronic medical record; ISOBAR: Identification, Situation, Observations, Background, Assessment/Action, Recommendation.

Further examination of individual ISOBAR elements identified differences in the discussion of clinical risks and clinical alerts (Figure 3).

#### Clinical Risks and Clinical Alerts Were Discussed

Handover of clinical risks and clinical alerts occurred more frequently in EMR-enabled wards. Clinical risks were discussed in 77% of EMR-enabled handovers compared with 60% in paper-based wards, while clinical alerts were discussed in 43% of EMR-enabled handovers compared with only 27% in the paper-based wards.

#### Bedside Handover

Handover occurred at the bedside more frequently in paper-based wards, with a mean compliance of 83%, compared with 63% in EMR-enabled wards.

#### Inclusion of the Patient, Family, or Caregiver

**Figure 4.**
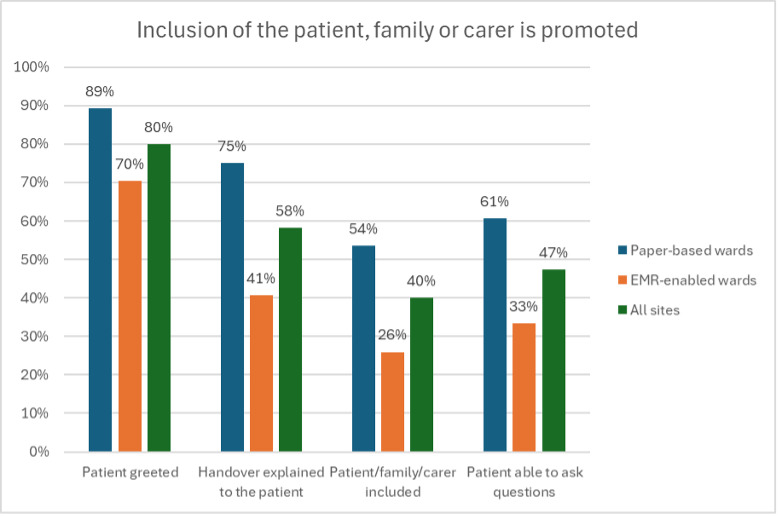
Inclusion of the patient, family, or caregiver is promoted. EMR: electronic medical record.

Promotion of patient, family, or caregiver inclusion occurred more frequently in paper-based wards. Compliance with all 4 patient inclusion indicators was observed in 70% of handovers in paper-based wards, compared with 43% in the EMR-enabled wards (Figure 4).

### Comparison With Organization-Wide Handover Audits

Observational audit findings were compared with results from Eastern Health’s Clinical Handover Nursing and Midwifery Audits conducted in 2022‐2023. Areas identified for improvement in the organization-wide audits aligned closely with findings in this case study, particularly in relation to bedside handover and patient and caregiver involvement.

Notably, overall performance relating to patient and caregiver inclusion was lower in this case study than in the organization-wide audits, suggesting potential variability in practice interpretation and reinforcing the need for further exploration of what constitutes effective patient inclusion and engagement in handover. Overall, these observational findings suggest that EMR availability was associated with variation in how handover was enacted across specific domains, rather than a uniform improvement or decline in overall handover quality.

### Posthandover Surveys

#### Overview

Analysis of the posthandover survey data commenced with the calculation of a composite score derived from the 5 Likert-scale questions assessing staff satisfaction, perceived handover quality, adherence to ISOBAR, accuracy, and comprehensiveness of handover. A maximum composite score of 25 was possible. Results for individual items and composite scores by cohort are presented in Tables6  7.

**Table 6.** Posthandover survey results (Likert scale questions). Overall score across all survey questions was 92% (median 97%) for paper-based and 91% (median 97%) for electronic medical record (EMR)-enabled handover**.**

Question	Paper-based mean (score)	Paper-based mean (%)	Paper-based median (score)	Paper-based median (%)	EMR-enabled mean (score)	EMR-enabled mean (%)	EMR-enabled median (score)	EMR-enabled median (%)
1. Are you satisfied with the handover you just received?	4.73	95	5	100	4.67	93	5	100
2. Rate the overall quality of the handover received	4.60	92	5	100	4.43	89	5	100
3. Did the handover follow the ISOBAR^a^ format?	4.40	88	5	100	4.53	91	5	100
4. Rate how accurate you believe the handover was?	4.80	96	5	100	4.70	94	5	100
5. Rate how comprehensive you believe the handover was?	4.60	92	5	100	4.47	89	5	100
Composite score (Q1-Q5)	23.1	93	23	92	22.8	91	23	92

a ISOBAR: Identification Situation Observations Background Assessment/Action Recommendation.

**Table 7.** Posthandover survey results (Closed questions). Overall score across all survey questions was 92% (median 97%) for paper-based and 91% (median 97%) for electronic medical record (EMR)-enabled handover**.**

Closed questions (Y/N)	Paper-based mean (%)	Paper-based median (%)	EMR-enabled mean (%)	EMR-enabled median (%)
6. Was the required content effectively communicated?	100	100	100	100
7. Were you given the opportunity to clarify information?	100	100	97	100
8. Was there any specific information you felt was missing? (count of no)	93	100	90	100
10. Was the duration of handover appropriate?	93	100	100	100
11. Was the inclusion of the patient, family, or caregiver promoted?	70	70	70	70

Composite scores demonstrated minimal difference between cohorts, indicating similar levels of staff satisfaction and perceived handover quality. Across 10 of the 11 survey questions, responses were overwhelmingly positive, with no mean scores falling below 88% in either cohort.

Staff perception of the promotion of patient, family, or caregiver inclusion was the only survey item demonstrating a notable reduction in perceived performance.

#### Staff Perception - Promotion of Patient Inclusion

While observed patient engagement during the observational audits was limited, staff-reported perceptions indicated higher levels of patient involvement during handover. Promotion of patient inclusion was recorded as equivalent across cohorts, with both paper-based and EMR-enabled wards reporting 70% affirmative responses to this survey item.

## Discussion

### Principal Findings

This case study examined the impact of EMR implementation on both the process and perceived quality of nursing handover. Particular attention was given to how digital information systems shape nurses’ access to, synthesis of, and communication about patient information. In this study, handover quality was operationalized using 2 complementary process-level measures: observed compliance with the organization’s Clinical Handover Standard, and nurses’ self-reported perceptions of handover quality following shift-to-shift handover exchange. Together, these measures provide insight into how EMR-mediated workflows influence handover behaviors, rather than downstream patient outcomes. Overall, EMR implementation did not uniformly improve handover quality; rather, its effects were heterogeneous across process domains, partially supporting the study objectives.

Quantitatively, overall compliance with the Clinical Handover Standard did not differ meaningfully between EMR-enabled and paper-based wards, nor did overall staff perceptions of handover quality. However, analysis of individual observational indicators revealed variation across specific domains. These included bedside location of handover, promotion of patient engagement, communication of clinical risks and alerts, handover duration, and frequency of interruptions. These findings suggest that while aggregate measures of handover quality appeared comparable, EMR implementation influenced *how* handover was enacted across specific components of the process.

### Observational Audits

#### EMR-Facilitated Handover Creates a Barrier to Performing Bedside Handover

Observational audit data demonstrated a lower frequency of bedside handover in EMR-enabled wards compared with paper-based wards.

Bedside handover encourages direct and real-time engagement with the patient [1]. Active patient participation in the process can provide immediate information on the patient’s condition [1]. Moreover, exchanging clinical information in the patient’s presence can assist in identifying patient concerns, promoting continuity, quality of care, and improved patient satisfaction [1]. Implementing bedside handover is therefore key to promoting and supporting patient engagement and participation in care.

In this study, the presence of the EMR appeared to act as a barrier to nurses performing bedside handover. This finding points to challenges associated with EMR implementation that may, in turn, reduce the level of patient engagement during handover. The following factors may help explain this finding.

#### Limitations of the Physical Environment

In the EMR-enabled wards in this case study, nurses access the EMR on mobile workstations via computers mounted onto purpose-built trolleys, commonly referred to as Workstations on Wheels (WOWs). While ergonomic and adequately mobile, these WOWs are still relatively large. Often observed during the observational audit was one nurse from the AM shift giving handover to 2 nurses on the PM shift. Frequently, both nurses receiving handover would each be interacting with their own WOW. At times, the nurse giving handover would also be accessing the EMR on their own WOW, though this appeared to occur less frequently, with the nurse giving handover most commonly referring to their own printed handover sheet, inclusive of hand-written notes.

Despite the EMR-enabled wards being located in a relatively new and modern facility with spacious patient rooms, the available physical space at the bedside remained insufficient for 3 nurses and 2 WOWs. The devices are bulky and difficult to maneuver. As a result, staff appeared reluctant to take the WOWs fully into patient rooms and were frequently observed remaining in or just outside the doorway. The lead researcher was also made aware that in some patient rooms within the EMR-enabled cohort, Wi-Fi connectivity was reduced, resulting in poor or limited EMR functionality at the bedside.

#### Social Barriers to Using Technology at the Bedside

In addition to these identified physical limitations, a more nuanced barrier is a social one, where the presence of the EMR at the patient’s bedside may be perceived as incongruous with a therapeutic environment. A positive and workable nurse-patient relationship develops when trust is established and maintained through an open exchange of information, supporting the delivery of interpersonal care [37,38]. In inpatient hospital settings, the nurse-patient relationship develops at the patient’s bedside. Use of active listening techniques during nurse-patient interactions, such as making and maintaining appropriate eye contact, facing the patient, and minimizing distractions [37,38], further contributes to this therapeutic connection. Introducing digital technology into the therapeutic bedside space may be perceived by nurses as intrusive. It may disrupt active listening practices and interfere with the development of the therapeutic relationship. The patient may therefore perceive that the information being shared is not valued.

Duffy et al [38] similarly note that EMR documentation at the point of care can distract nurses’ attention from the patient. This may reduce communication quality, appropriate eye contact, and overall meaningful interaction [38]. Misto [39] explored the impact of electronic documentation in the patient’s presence on the nurse-patient relationship and noted that nursing staff perceptions of the EMR’s influence on the therapeutic relationship were mixed [39]. Positive impacts included enhanced access to data at the patient’s bedside and the ability to document contemporaneously and more efficiently, resulting in more time to spend with the patient [39]. Negative impacts centered on the recurring premise that using the EMR at the bedside is a distraction, resulting in both ‘missed opportunities to connect with patients,’ and an increased likelihood that subtle, nonverbal cues could be missed [39]. This was attributed to nurses being preoccupied with interacting with the EMR rather than being fully attentive to the patient [39]. Nurses in that study also reported that patients felt less valued when this occurred [39].

Gaudet [40] also explored the changes in nurse-patient interactions associated with the introduction of EMR documentation at the bedside, noting that this may create “an automatic, machine-like, task centered bedside environment” [40].

It is therefore possible that nurses feel that the insertion of a large and bulky, potentially distracting piece of digital technology into the space at the patient’s bedside may negatively impact the therapeutic relationship, serving only to make handover more impersonal rather than enhance it.

#### Cognitive Barriers to Performing EMR-Facilitated Handover at the Bedside

A recent review article exploring the impact of an EMR implementation on nursing handover determined that existing EMR interfaces are not currently meeting the needs of nurses during handover [22]. Navigating the EMR has been shown to impact cognitive workload due to the range of tasks required of users [26]. Combined with the way information is presented in the EMR, this results in fragmented or siloed data that must be skimmed through, sorted, and deciphered [18,24,25]. In this study, nurses were frequently required to navigate multiple EMR screens to locate alerts, risks, and clinical updates, reflecting well-recognized information fragmentation within EMR systems [25]. In this context, fragmentation shifts cognitive effort away from clinical reasoning and toward system navigation [25,41].

Handover is already a cognitively challenging task, requiring the nurse to be able to swiftly analyze and synthesize patient data across multiple information sources [24,42,43] and engage in complex knowledge-sharing processes [18]. The expectation that this demanding task also occurs at the bedside, involves the patient, and is mediated through an EMR that is often difficult to navigate, may further increase this burden. This raises an important question: Does this combination unintentionally work against the goal of improving handover quality and patient engagement? Is the presence of the patient themselves now an additional barrier to achieving the desired level of handover quality? Gaudet [40] explores this conundrum where ‘the complexity of the patients, the computerized prioritisations of tasks and the interruptions, limited the nurses’ ability to nurture and become familiar with their patients, thus creating “an environment that does not put the patients’ concerns at the center of care” [40].

From an informatics perspective, EMR-mediated handover requires nurses to simultaneously navigate, prioritize, and synthesize information. At the same time, they must maintain effective interpersonal communication with both colleagues and patients. Together, these demands increase cognitive workload during an already complex task. Given that a fully optimized EMR solution for nursing has not yet been realized [22], nurses may be reluctant to bring EMR-facilitated handover to the bedside. Doing so may be perceived as increasing complexity and potentially compromising handover quality.

#### Patient Engagement Was Better Promoted in Paper-Based Environments

Where a reduced frequency of bedside handover was observed in the EMR-enabled cohort, so too were lower levels of patient engagement and decreased promotion of patient, family, and caregiver inclusion in handover. Although the compliance gap for initially greeting the patient was smaller between the cohorts, meaningful patient inclusion cannot occur when handover takes place away from the bedside. This was demonstrated by the widening performance gaps for the remaining patient engagement indicators, ”Including the Patient in Handover” and “Providing the Patient an Opportunity to Ask Questions.”

Reduced frequency of bedside handover in the EMR-enabled wards was therefore identified as a stand-alone barrier to promoting patient engagement as measured by observational indicators of inclusion and the opportunity to ask questions.

#### Other Known Barriers to Bedside Handover and Patient Engagement

Despite the known benefits of bedside handover in improving patient engagement and supporting patient-centered care, studies continue to elucidate reluctance from nurses to perform handover at the bedside. Independent of EMR introduction, commonly cited barriers to bedside handover include concerns about patient privacy and confidentiality [44-46]. Nurses also report worries about being asked difficult questions or appearing unprofessional in front of patients [44,45]. Additional concerns relate to perceived prolongation of handover [47,48] and disruption to communication flow [46,47].

A pre-existing resistance to bedside handover for these myriad reasons must therefore be considered as a contributing factor to the gap in bedside handover implementation across both cohorts in this study. Where an existing baseline gap in practice may already be evident, the introduction of the EMR, and all the additional potential barriers it brings, has possibly further compounded this issue in the EMR-enabled wards, making bedside handover even less achievable.

#### Handover of Critical Information Occurred More Readily in EMR-Enabled Wards

In the hospital environment, EMRs are accessed by clinicians throughout the course of a patient’s treatment, replacing bulky paper-based medical records. In this case-study context, an EMR was introduced and mobile WOWs provided, providing up-to-date clinical information at the point of care and during handover. An EMR facilitates real-time sharing of patient data and has the potential to improve efficiency and care coordination [49]. The results of the observational audit support this, with improved handover of critical information such as clinical alerts and clinical risks evident in the EMR-enabled wards compared with the paper-based cohort.

This also aligns with marginally better performance observed for overall ISOBAR compliance in the EMR-enabled wards, where more consistent practice in using the mnemonic may have contributed to the improved handover of risks and alerts.

#### Reliance on Other Tools to Support Handover

In paper-based environments in this case study, nurses predominantly rely on printed handover sheets generated by bed management software known as Patient Flow Manager (PFM). PFM is not linked to the organization’s EMR and clinical information stored within it, and it does not form part of the medical record.

To populate these handover sheets, clinical information is transcribed from the scanned patient history or EMR and manually entered into PFM. Nursing staff add and update clinical information in PFM throughout their shift, including the removal of any outdated information. It is not routinely used by medical or allied health staff in this way. Unfortunately, a reliance on duplication and manual data entry processes has the potential to result in transcription errors, decreasing the accuracy of the information contained on these handover sheets. Conversely, the EMR is considered the source of truth for clinical patient information, inclusive of clinical alerts and clinical risks, which have been configured to provide a visual alert to clinicians.

In the EMR-enabled wards, nurses also use PFM and are provided with PFM-generated printouts at the commencement of their shifts. In these wards, however, the EMR is always accessible during handover and is frequently used to check information, most notably by the handover recipients. This is consistent with recent studies, which predominantly described EMR use during handover as verifying or double-checking information [24,25,41] and keeping up to date with the latest developments [18].

In paper-based environments, nurses rely heavily on printed handover sheets. The information on these sheets may be outdated, incomplete, or inaccurate. Where critical changes in the patient’s condition occur, it is imperative that nurses are immediately aware of changes to any clinical alerts and clinical risks. This will ensure that the most appropriate care, interventions, and safety precautions can be implemented to promote patient and staff safety.

Persistent reliance on PFM-generated paper handover sheets was observed in both cohorts, including the EMR-enabled wards. In this study, this pattern signaled a socio-technical misalignment between the EMR design and nursing handover workflows. Despite the EMR being the authoritative source of clinical information, nurses continued to depend on nonintegrated artifacts to support their cognitive load, information sequencing, and real-time note-taking during handover. This behavior suggests that existing EMR interfaces did not adequately support nurses’ information needs during handover, reinforcing findings that poor task–technology fit drives workarounds and partial adoption of digital systems [22].

#### Handover Was More Efficient in the EMR-Enabled Wards

Observational audit data demonstrated reduced median handover duration in EMR-enabled wards compared with paper-based wards. Performance was also more consistent in the EMR-enabled environments, with more variability of duration noted in the paper-based wards.

Across a number of studies, accessing and navigating the EMR has been described as time-consuming [24,41], cumbersome [41], arduous [24], and not necessarily able to provide pertinent information quickly [43].

Despite these potentially negative connotations, studies reporting specific impacts of EMR implementation on handover duration offered mixed results. Hertzum and Simonsen [50] sought to understand the impact of a trial EMR implementation, finding that there was no significant difference in handover duration postintervention [50]. Correspondingly, Alghenami’s [51] 2012 study exploring the role of EMRs in structuring handover communications found that nurses did not identify the EMR as a factor that would impact handover duration [51]. There is growing evidence, however, that use of specific EMR-structured handover tools or EMR-generated printouts can assist in making handover quicker and more efficient [52-54]. It is important to note, nonetheless, that positive outcomes are highly contingent on whether these tools are adequately aligned with the nature of the clinical work [55] and offer sufficient functionality to support the task [56]. Conversely, where an EMR interface does not adequately suit the task at hand, it fails the end user, which may result in incomplete adoption of the new technology and a continued reliance on paper-based tools [22]. Ultimately, when a gap in functionality or design is not readily addressed, or poor user experience persists, this has the potential to lead to user frustration and even boycott [56].

At Eastern Health, in the EMR-enabled wards, nurses were observed navigating various parts of the EMR during handover, yet they did not use any specific EMR-structured handover tools or EMR-generated printouts. Further to this, nurses across both cohorts were almost always observed consulting or making handwritten notes on paper handover sheets printed from PFM. Nurses in the EMR-enabled cohort demonstrated willingness to adopt EMR use during handover. However, the continued reliance on PFM-generated printouts suggests a gap in EMR design or functionality. While the PFM-generated printouts clearly provide support to nurses during handover, they remain problematic as they do not draw clinical information directly from the EMR. Ideally, an EMR-generated printout would replace those generated via PFM and thus provide the most up-to-date clinical information. Ultimately, these printouts must be purposefully designed by and for nurses in order that they fully support the task being performed.

#### Handover Occurring in EMR-Enabled Wards Experienced Fewer Interruptions

The paper-based cohort experienced more interruptions during handover, noting this may well be an anomaly given the very small sample size. Current research on the impact of interruptions during EMR use appears limited to those that occur via the EMR itself, such as automated alerts, reminders, and other clinical decision support mechanisms [57-59], or broader workflow interruptions associated with EMR implementations in general [60-62]. This case study sought to measure external interruptions caused by patients, phones, alarms, or other staff in the ward environment. The reason for fewer interruptions occurring in the EMR-enabled cohort is, therefore, entirely speculative.

A very well-researched field that could offer some useful comparisons is the frequency and impact of interruptions on medication administration workflows, which have the potential to result in medication errors. As a known high-risk activity, efforts to prevent and reduce interruptions that may lead to errors during medication rounds have also been thoroughly explored. Frequently used strategies to reduce and manage the risk of interruptions include the use of standardized checklists and protocols such as the 5 rights of medication administration, quiet zones for medication preparation, diversion strategies, and visual alerts such as wearable sashes and vests asking that others “Do Not Disturb” and “Do Not Interrupt” [63-65]. Nurses are therefore highly aware of this increased risk to patient safety, noting also that these types of interruptions are significant contributing factors to increasing mental workloads in hospital work environments [66]. One possible explanation is that engagement with the EMR may provide a visible cue to other staff that nurses are occupied with handover, potentially reducing external interruptions in a manner similar to visual “Do No Disturb” signals used during medication administration.

These findings should therefore be interpreted as indicative process trends rather than definitive performance improvements, and further investigation with larger samples or longitudinal designs may clarify their significance.

### Staff Perception of Patient Engagement

Observational audit findings relating to patient engagement during nursing handover did not correlate with staff’s self-reported perceptions of their own efforts to promote patient participation.

While bedside handover alone is insufficient to ensure meaningful patient engagement in the process [45], it remains a fundamental prerequisite in order for patient participation to occur. In this study, observational audits identified a compliance gap with bedside handover across both cohorts, with this gap more significant in the EMR-enabled wards. On this basis, lower observed rates of bedside handover in the EMR-enabled cohort would be expected to correspond with similarly reduced rates of self-reported promotion of patient engagement during handover. And yet the staff survey found that this was occurring in equal proportions across both cohorts, around 70% of the time.

The large mixed methodology study of Street et al [45] examining patient participation in handover may assist in explaining this, observing that whilst nurses do perceive the value in patient participation, ‘the best way to effectively help patients be more involved in bedside nursing handover is poorly understood’ [45]. The findings of this case study support this notion, suggesting that nurses, who may not understand how to effectively engage patients, may have limited insight into whether their handover practices genuinely facilitate patient engagement and participation.

This discrepancy may reflect an overestimation of patient engagement in staff self-reported data, potentially influenced by social desirability bias, whereby participants provide responses and report practices that are perceived as more acceptable [67]. In this instance, the self-reported practice aligned better with organizational expectations and the requirements of the Clinical Handover Standard, despite the decreased incidence of bedside handover necessary to facilitate the desired engagement.

Collectively, these findings highlight the value of using multiple measurement approaches when evaluating handover quality, as reliance on self-report alone may overestimate adherence to patient engagement principles.

### Staff Satisfaction With Handover Quality

Despite the reduced occurrence of bedside handover, potential data fragmentation, workflow changes, and reliance on workarounds (including paper-based and nonintegrated artifacts) in EMR-enabled wards, staff satisfaction with overall handover quality remained high across both cohorts. Clinicians may report satisfaction with systems they have learned to cope with, even when those systems impose additional cognitive or workflow burdens [56]. These findings likely reflect nurses’ ability to adapt to existing system constraints rather than optimal alignment between EMR design and nursing handover workflows.

Taken together, these findings suggest that staff satisfaction alone is an insufficient indicator of EMR effectiveness, reinforcing the need to examine how digital systems shape cognitive workload, workflow integration, and communication practices during nursing handover.

The contrast between consistently high staff satisfaction and observed variability in handover behaviors suggests adaptation and normalization of system constraints, reinforcing the importance of examining both objective and perceptual measures when evaluating digital clinical systems. Integrating observational and perceptual data strengthened interpretive validity and enabled identification of important differences between enacted and perceived practice that would not have been apparent using a single measurement approach.

### Limitations

Several limitations should be considered when interpreting the findings of this study.

First, the relatively small sample size of 60 handovers limited the ability to detect statistically significant differences across all measures, particularly given the complexity and variability inherent in clinical handover practices. While sampling 5 handovers per ward enabled exposure to routine practice within each setting, the number of observations per cohort was insufficient to support more granular analyses or make more robust comparisons. As a result, findings should be interpreted as indicative of patterns of use and areas of variation rather than definitive estimates of practice prevalence. In addition, this study focused on process-level indicators of nursing handover quality, including observed compliance with the organization’s Clinical Handover Standard and nurses’ self-reported perceptions of handover quality. These measures provide important insights into how handover is conducted in different environments but do not directly assess patient outcomes or safety events. As such, findings should be interpreted as reflecting differences in handover processes only. Furthermore, as an exploratory case study conducted within a single health service, the findings have limited generalizability beyond similar organizational and hybrid EMR contexts. However, the design provides rich, practice-based insight into how nursing handover is shaped within real-world digital environments.

Second, the use of a single auditor introduces the potential for investigator bias. To mitigate this risk, the observational audit tool was designed to minimize subjectivity, with items structured wherever possible to limit reliance on auditor interpretation. Consistent use of a single trained auditor also supported standardization of data collection across sites.

Third, a degree of Hawthorne effect may have occurred, as participants were aware that they were being observed. This is particularly relevant for highly visible elements of the Clinical Handover Standard, such as conducting handover at the bedside and performing patient identification checks. While this may have influenced overall adherence to the Clinical Handover Standard, it is unlikely to have affected the comparative analysis between the 2 cohorts, as any observation effect would have been equally probable in both groups.

Finally, the study focused exclusively on shift-to-shift nursing handover within inpatient wards and did not include handovers occurring in other settings such as interdepartmental transfers and other transitions of care. Future research should consider observing and evaluating handovers across a broader range of clinical contexts, with particular attention to how EMRs are configured and used to facilitate and support nursing handover under varying workflow, environmental, and cognitive demands.

### Conclusions

This case study demonstrates that EMR implementation has the potential to produce both beneficial and unintended effects on the quality of shift-to-shift nursing handovers. While EMR-enabled wards showed positive impacts in accessing and communicating up-to-date clinical information, particularly in relation to clinical alerts and risks, these benefits did not translate uniformly across all dimensions of handover quality.

Findings indicate that a pre-existing reluctance to perform bedside handover may be exacerbated in EMR-enabled environments, with negative implications for patient inclusion and engagement. Limitations related to the physical environment, EMR interface and design, social factors, and cognitive factors appear to impede nurses’ ability to consistently promote patient participation during handover. These findings suggest that nurses may not have a clear or comprehensive understanding of the actions necessary to actively promote patient engagement, nor how to effectively assess these efforts, a challenge that appears more pronounced in digitally mediated handover contexts. This suggests a gap in informatics-enabled support for nursing practice, particularly in translating digital handover processes into patient-centered behaviors.

Positive impacts of EMR implementation included improved efficiency and reduced interruptions during handover, indicating a degree of user acceptance and adaptation. However, continued reliance on paper-based PFM-generated handover forms highlights persistent gaps in EMR functionality or design, and the need for workarounds to support cognitive and workflow demands during handover. While this study did not examine patient or safety outcomes directly, it provides important process-level evidence to inform the optimization of nursing handover within digitally enabled environments.

Collectively, these findings indicate that the presence of an EMR alone is insufficient to ensure high-quality nursing handover. Rather, the impact of EMR implementation on handover quality is mediated by nursing informatics capability and the degree to which digital systems are designed and aligned with nurses’ cognitive processes and clinical workflows. Where EMR interfaces fragment information or fail to provide integrated, task-specific handover views, nurses compensate through workarounds that may limit the potential benefits of digital systems. Optimizing nursing handover in digitally enabled environments requires EMR designs that explicitly support handover as a complex, cognitively demanding, and safety-critical nursing activity, rather than assuming that quality improvements will occur through digitization alone.

### Future Research

Future research should examine in greater depth how nurses interact with EMR systems during handover, including patterns of use, information navigation, and reliance on workarounds. Detailed analysis of user interaction with the EMR would assist in determining the degree to which the current technology suits the task being undertaken and which current digital tools are suited to the cognitive and workflow demands of nursing handover.

Qualitative exploration of nurses’ perceived barriers and facilitators to EMR use during handover would further inform understanding of how EMR interface design, functionality, workflow integration, and device availability may influence handover practices. Such insights could support the identification of design flaws that contribute to the ongoing reliance on paper-based handover forms and other nonintegrated artifacts in EMR-enabled environments.

Future studies may benefit from the evaluation of discrete components of EMR implementation, including device footprint, software interface design, workflow integration, and overall user experience, to better understand each element’s relative impact on the quality and effectiveness of nursing handover.

Comparative analyses across wards or sites with differing levels of digital maturity, including electronic prescribing and medication administration, may offer further clarification as to how specific EMR functionalities influence nursing handover processes and outcomes.

## Supplementary material

10.2196/85909 Multimedia Appendix 1 Eastern Health clinical handover standard.

## Abbreviations

CFS CRGC: Communicating for Safety Clinical Risk Governance Committee
EMR: electronic medical record
ISOBAR: Identification Situation Observations Background Assessment/Action Recommendation
NSQHS: National Safety and Quality Health Service
PFM: Patient Flow Manager
WOW: Workstations on Wheels

## References

[1] Slade D, Murray KA, Pun JKH, Eggins S (2019). Nurses’ perceptions of mandatory bedside clinical handovers: an Australian hospital study. J Nurs Manag.

[2] Chapman YL (2016). Nurse satisfaction with information technology enhanced bedside handoff. Medsurg Nurs.

[3] Dunsford J (2009). Structured communication: improving patient safety with SBAR. Nurs Womens Health.

[4] Jorm CM, White S, Kaneen T (2009). Clinical handover: critical communications. Med J Aust.

[5] ACo S, QiH C (2012). Safety and Quality Improvement Guide Standard 6: Clinical Handover.

[6] Patterson ES, Wears RL (2010). Patient handoffs: standardized and reliable measurement tools remain elusive. Jt Comm J Qual Patient Saf.

[7] Johnson M, Sanchez P, Zheng C (2016). Reducing patient clinical management errors using structured content and electronic nursing handover. J Nurs Care Qual.

[8] Ong MS, Coiera E (2011). A systematic review of failures in handoff communication during intrahospital transfers. Jt Comm J Qual Patient Saf.

[9] Yee KC, Wong MC, Turner P (2009). “HAND ME AN ISOBAR”: a pilot study of an evidence-based approach to improving shift-to-shift clinical handover. Med J Aust.

[10] Sujan M, Spurgeon P, Cooke M (2015). The role of dynamic trade-offs in creating safety—a qualitative study of handover across care boundaries in emergency care. Reliab Eng Syst Saf.

[11] Clark JR, Stanton NA, Revell KMA (2019). Identified handover tools and techniques in high-risk domains: using distributed situation awareness theory to inform current practices. Saf Sci.

[12] Milesky JL, Baptiste DL, Shelton BK (2018). An observational study of patient handover communications among nurses on an oncology critical care unit. Contemp Nurse.

[13] Haig KM, Sutton S, Whittington J (2006). SBAR: a shared mental model for improving communication between clinicians. Jt Comm J Qual Patient Saf.

[14] Stanton NA, Salmon PM, Walker GH, Salas E, Hancock PA (2017). State-of-science: situation awareness in individuals, teams and systems. Ergonomics.

[15] (2021). National Safety and Quality Health Service Standards.

[16] Chaboyer W, McMurray A, Wallis M (2010). Bedside nursing handover: a case study. Int J Nurs Pract.

[17] Forde MF, Coffey A, Hegarty J (2020). Bedside handover at the change of nursing shift: a mixed-methods study. J Clin Nurs.

[18] Ihlebæk HM (2020). Lost in translation - silent reporting and electronic patient records in nursing handovers: An ethnographic study. Int J Nurs Stud.

[19] Jimma BL, Enyew DB (2022). Barriers to the acceptance of electronic medical records from the perspective of physicians and nurses: a scoping review. Inform Med Unlocked.

[20] Silow-Carroll S, Edwards JN, Rodin D (2012). Using electronic health records to improve quality and efficiency: the experiences of leading hospitals. Issue Brief (Commonw Fund).

[21] Gatiti P, Ndirangu E, Mwangi J, Mwanzu A, Ramadhani T (2021). Enhancing healthcare quality in hospitals through electronic health records: a systematic review. J Health Inform Dev Ctries.

[22] Browning L, Raza-Khan U, Leggat S, Boyd JH (2025). The impact of electronic medical record implementation on the process and outcomes of nursing handover: a rapid evidence assessment. J Nurs Manag.

[23] Boonstra A, Versluis A, Vos JFJ (2014). Implementing electronic health records in hospitals: a systematic literature review. BMC Health Serv Res.

[24] Staggers N, Clark L, Blaz JW, Kapsandoy S (2011). Why patient summaries in electronic health records do not provide the cognitive support necessary for nurses’ handoffs on medical and surgical units: insights from interviews and observations. Health Informatics J.

[25] Lindroth HL, Pinevich Y, Barwise AK (2022). Information and data visualization needs among direct care nurses in the intensive care unit. Appl Clin Inform.

[26] Wisner K, Lyndon A, Chesla CA (2019). The electronic health record’s impact on nurses’ cognitive work: an integrative review. Int J Nurs Stud.

[27] Van Der Meijden MJ, Tange HJ, Troost J, Hasman A (2003). Determinants of success of inpatient clinical information systems: a literature review. J Am Med Inform Assoc.

[28] Goodhue DL (1995). Understanding user evaluations of information systems. Manage Sci.

[29] Graves JR, Corcoran S (1989). The study of nursing informatics. Image (IN).

[30] Staggers N, Gassert CA, Curran C (2002). A Delphi study to determine informatics competencies for nurses at four levels of practice. Nurs Res.

[31] Woods L, Cummings E, Dobroff N (2021). Healthier Lives, Digitally Enabled.

[32] Darvish A, Bahramnezhad F, Keyhanian S, Navidhamidi M (2014). The role of nursing informatics on promoting quality of health care and the need for appropriate education. Glob J Health Sci.

[33] Mather C, Cummings E, Gale F (2019). Nurses as stakeholders in the adoption of mobile technology in Australian health care environments: interview study. JMIR Nurs.

[34] Yin RK (2009). Case Study Research: Design and Methods.

[35] Creswell JW, Poth CN (2016). Qualitative Inquiry and Research Design: Choosing among Five Approaches.

[36] Morgan SJ, Pullon SRH, Macdonald LM, McKinlay EM, Gray BV (2017). Case study observational research: a framework for conducting case study research where observation data are the focus. Qual Health Res.

[37] Arnold EC, Boggs KU (2019). Interpersonal Relationships E-Book: Professional Communication Skills for Nurses.

[38] Duffy WJ, Kharasch MS, Du H (2010). Point of care documentation impact on the nurse-patient interaction. Nurs Adm Q.

[39] Misto K, Padula C, Bryand E, Nadeau K (2019). Nurses’ perception of the impact of electronic documentation on the nurse-patient relationship. J Nurs Care Qual.

[40] Gaudet CA (2016). Electronic documentation and nurse-patient interaction. ANS Adv Nurs Sci.

[41] Ghosh K, Dohan MS, Curl E, Goodwin M, Morrell P, Guidroz P (2022). Information tools for care coordination in patient handover: is an electronic medical record enough to support nurses?. Health Care Manage Rev.

[42] Staggers N, Clark L, Blaz JW, Kapsandoy S (2012). Nurses’ information management and use of electronic tools during acute care handoffs. West J Nurs Res.

[43] Galatzan BJ, Carrington JM (2018). Exploring the state of the science of the nursing hand-off communication. CIN.

[44] Gregory S, Tan D, Tilrico M, Edwardson N, Gamm L (2014). Bedside shift reports: what does the evidence say?. J Nurs Adm.

[45] Street M, Dempster J, Berry D (2022). Enhancing active patient participation in nursing handover: a mixed methods study. J Clin Nurs.

[46] Tobiano G, Whitty JA, Bucknall T, Chaboyer W (2017). Nurses’ perceived barriers to bedside handover and their implication for clinical practice. Worldviews Evid Based Nurs.

[47] Drach-Zahavy A, Goldblatt H, Maizel A (2015). Between standardisation and resilience: nurses’ emergent risk management strategies during handovers. J Clin Nurs.

[48] Malfait S, Eeckloo K, Van Biesen W, Van Hecke A (2019). Barriers and facilitators for the use of NURSING bedside handovers: implications for evidence-based practice. Worldviews Evid Based Nurs.

[49] Wills MJ, El-Gayar OF, Sarnikar S (2011). Beyond meaningful use: a model for evaluating electronic health record success.

[50] Hertzum M, Simonsen J (2008). Positive effects of electronic patient records on three clinical activities. Int J Med Inform.

[51] Alghenaimi S (2012). The Role of Electronic Health Records in Structuring Nursing Handoff Communication and Maintaining Situation Awareness.

[52] Nickel N, Amin D, Shakeel F, Germain A, Machry J (2021). Handoff standardization in the neonatal intensive care unit with an EMR-based handoff tool. J Perinatol.

[53] Panda S (2020). Nursing shift handoff process: using an electronic health record tool to improve quality. CJON.

[54] Vawdrey DK, Stein DM, Fred MR, Bostwick SB, Stetson PD (2013). Implementation of a computerized patient handoff application. AMIA Annu Symp Proc.

[55] Bergey MR, Goldsack JC, Robinson EJ (2019). Invisible work and changing roles: Health information technology implementation and reorganization of work practices for the inpatient nursing team. Soc Sci Med.

[56] Ammenwerth E, Iller C, Mahler C (2006). IT-adoption and the interaction of task, technology and individuals: a fit framework and a case study. BMC Med Inform Decis Mak.

[57] Oluoch T, Santas X, Kwaro D (2012). The effect of electronic medical record-based clinical decision support on HIV care in resource-constrained settings: a systematic review. Int J Med Inform.

[58] Powers EM, Shiffman RN, Melnick ER, Hickner A, Sharifi M (2018). Efficacy and unintended consequences of hard-stop alerts in electronic health record systems: a systematic review. J Am Med Inform Assoc.

[59] Todd B, Shinthia N, Nierenberg L, Mansour L, Miller M, Otero R (2021). Impact of electronic medical record alerts on emergency physician workflow and medical management. J Emerg Med.

[60] Gephart S, Carrington JM, Finley B (2015). A systematic review of nurses’ experiences with unintended consequences when using the electronic health record. Nurs Adm Q.

[61] Lin A, Harris M, Zalis M (2010). Initial observations of electronic medical record usage during CT and MRI interpretation: frequency of use and impact on workflow. AJR Am J Roentgenol.

[62] Makoul G, Curry RH, Tang PC (2001). The use of electronic medical records: communication patterns in outpatient encounters. J Am Med Inform Assoc.

[63] Raban MZ, Westbrook JI (2014). Are interventions to reduce interruptions and errors during medication administration effective?: a systematic review. BMJ Qual Saf.

[64] Relihan E, O’Brien V, O’Hara S, Silke B (2010). The impact of a set of interventions to reduce interruptions and distractions to nurses during medication administration. Qual Saf Health Care.

[65] Yoder M, Schadewald D, Dietrich K (2015). The effect of a safe zone on nurse interruptions, distractions, and medication administration errors. J Infus Nurs.

[66] Kim JH, Parameshwara N, Guo W, Pasupathy KS (2019). The impact of interrupting nurses on mental workload in emergency departments. Int J Hum Comput Interact.

[67] Matthews BA, Baker F, Spillers RL (2003). How true is true? assessing socially desirable response bias. Qual Quant.

