# Supplementary material for: Exploring the Impact of Electronic Medical Record–Enabled Versus Paper-Based Systems on the Quality of Nursing Handover: Comparative Case-Study
Source: JMIR Nurs. 2026 May 12;9:e85909. doi: 10.2196/85909 (PMC13168791; doi:10.2196/85909)

**Multimedia Appendix - Eastern Health Clinical Handover Standard (as at November 2022)**

**Clinical Handover Standard**

1. Standard

The overarching purpose of this standard is to ensure optimal clinical communication occurs within, and between all disciplines and departments, throughout the care continuum, to optimise patient safety and outcomes.

Clinical handover is the “transfer of professional responsibility and accountability for some or all aspects of care for a patient, or groups of patients to another person or professional group on a temporary or permanent basis” (Australian Commission on Safety and Quality in Healthcare, 2011).

Eastern Health recognises the importance of clinical handover in the delivery of safe, effective, high quality care.

Eastern Health Clinical Handovers;

• Are undertaken using a consistent and structured process (ISOBAR – see Attachment 1) which includes a minimum data set

• Recognise that clinical handover requires effective communication between clinicians, including an opportunity to clarify information

• Maintain the patient’s right to confidentiality and privacy - sharing of information is based upon the relevance and impact to care and outcomes

• Promote inclusion of patients and where appropriate their carers whilst acknowledging that sensitivity is required where other patients or visitors may overhear patient information

• Consider the need to engage interpreters if patients have communication difficulties such as cultural and linguistic diverse backgrounds or hearing impairments

• Occur prior to, or at the time when clinician/s transfer care and accountability and acknowledges the transfer of accountability, for some or all of the patient’s care

• Ensure adequate preparation prior to undertaking a clinical handover, making certain that the process is efficient and that all relevant information is transferred

• The clinician providing direct care leads the clinical handover, and where possible, handover occurs as face-to-face communication

• Within mental health settings a team handover occurs prior to individual handover to ensure all staff are aware of the current milieu and risk issues present within the unit

• Respect the importance of handover, with minimal interruptions and/or distractions

• Documentation of the handover is recorded within the progress notes, or equivalent, in accordance with the Clinical Documentation Standard

New Eastern Health clinical staff will be orientated to Eastern Health’s clinical handover systems.

Clinical incidents relating to clinical handover are reported and investigated as per the Incident Reporting, Investigation and Management Standard.

2. Definition of terms

Patient: For the purposes of this policy, the word patient can mean client, consumer or resident who is in receipt of clinical care provided by Eastern Health.

ISOBAR: The framework used at Eastern Health to support structured clinical handover.

I – Introduction/ Identity, S – Situation, O – Observations, B – Background, A – Assessment, R – Request / Recommendations

Intra-Hospital Clinical Handover /Transfer: refers to the handover of patients/consumers within the same geographical location. This includes handover due to a change of ward, department or unit. The patient transfer may be temporary or permanent.

Inter-Hospital Clinical Handover/Transfer: refers to the handover of patients/consumers to a different geographical location (i.e. another site, facility, hospital). This type of handover may be between Eastern Health sites or external to Eastern Health (i.e. transfers to other facilities; transfer to and from a community provider). For nursing transfers between Eastern Health sites, wards or ambulatory care, the ISOBAR Handover form (EH 401510) should be utilised. For patients exiting via transit lounge or to an external care provider (other public hospital, private health facility or residential aged care), nursing transfer form ‘Non Eastern Health ISOBAR handover, should be used). More information on the requirements for clinical handover at time of patient transfer can be found in the ‘Clinical Handover Transfer Practice Guideline.’ Note: transfer forms are not required for time critical patients. ICU transfers will use an ICU ISOBAR transfer form.

Daily huddle/planning huddle/Multidisciplinary Team Meeting: a short multidisciplinary meeting focusing on addressing / escalating barriers to discharge and allocating staff to arrange the required elements to facilitate discharge. The daily huddle will also identify patients at risk of harm (i.e. delirium, falls, pressure injuries, fasting) or patients who have had new clinical issues in the preceding 24hrs.

3. Name of Policy to which Standard relates

Safe and High Quality Care

4. Associated Guidelines / Procedures

Transfer/Discharge Standard for Bed Based Services (2908)

Clinical Documentation Standard (203)

Clinical Handover Transfer Practice Guideline (2341)

Nursing and Midwifery Practice Guideline (2283)

Daily Operating System (3237)

Patient Journey, Communication and Welcome Board (2415)

5. Processes

5.1 Settings and context for clinical handover

Clinical handover occurs in a variety of settings and contexts throughout the health service and across a patient’s continuum of care including:

• Shift to shift or within a shift

o Intra professional, e.g. nurse to nurse, doctor to doctor, including covering doctors, physio to physio, case manager to case manager

o Multi-disciplinary (e.g. clinical huddles, multidisciplinary team meetings)

• Escalation of deteriorating patient

• Intra-hospital patient transfers including from one ward to another ward, from a diagnostic, procedural area or specialist consultation area

• Inter-hospital patient transfers

o Transfer to another Eastern Health site (acute to subacute, between acute facilities, transition care program, Eastern Health RACFs)

o Patient transfers from bed-based to Eastern Health ambulatory or community based services (e.g. Post-Acute Care, Hospital Admission Risk Program (HARP), Sub acute Ambulatory Care services, ED to ambulatory services)

o Transfer external to Eastern Health (other Public Health Service, Private Hospital, Residential Aged Care Facility)

o Patient transfers to and from an external community provider (General Practitioner, Community Health including Mental Health services, Private Medical Specialist)

5.2 Preparation for clinical handover

a. Leadership – a leader is nominated for each clinical handover

b. Embedded in daily work – clinical handover is valued and an essential part of daily work. Clinical staff are available to participate in the handover of all patients relevant to them.

c. Handover participants – are identified and orientated to the clinical handover process when commencing in a new work area. Handover participants:

• Reflect the multidisciplinary team where possible and relevant

• Patients and carers should be recognised and included whenever possible as handover participants.

• Clinical handover participants are involved in regular review of clinical handover processes

d. Time – where clinical handover occurs as part of scheduled daily work (i.e. shift to shift, daily huddles) clinical handover occurs at an agreed frequency, at a set time and for an agreed duration. Clinical areas should embed local strategies to reinforce punctuality and duration.

e. Location – a set location should be identified for handover to occur. Where possible, clinical handover will occur face to face and in the patient’s presence (bedside handover).

Multidisciplinary huddles should occur in a location where clinical staff have access to the electronic Patient Journey Board.

5.3 Handover Process

Clinical Handovers at Eastern Health will utilise the ISOBAR framework. The way in which this framework is applied may vary depending on the context and setting for clinical handover and allows for flexible standardisation.

The minimum required information for clinical handovers using the ISOBAR Framework is:


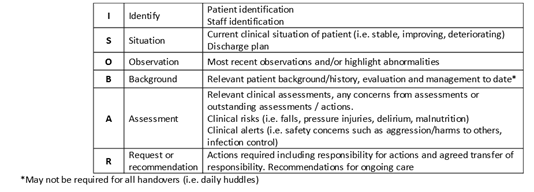


At the completion of clinical handover, the designated handover leader and those participating / receiving handover should:

o Check that those receiving handover understand the key issues

o Ensure there is agreement on outstanding assessments / actions / tasks and the allocation of these to teams members

o The transfer of responsibilities for ongoing care is acknowledged

5.4 Clinical handover documentation

Clinical Handover is documented according to the Clinical Documentation Standard.

Documentation of clinical handover in the medical record / electronic medical record is required where there is transfer of patient care (i.e. nursing shift to shift, inter hospital, intra hospital transfer or discharge). The exception to this is medical shift – shift handover (within the same or covering clinical unit). Medical shift-shift handover will occur using the ISOBAR structure and the responsibility for medical care of the patient is transferred for the period of time until the next handover to the treating medical team.

Documentation of clinical handover in the medical record can be completed by the clinician receiving or providing handover and will include: the date/time of handover, the person transferring responsibility of care, the person accepting responsibility of care and acknowledgement that handover was provided following the ISOBAR structure.

5.5 Clinical handover in the setting of a deteriorating patient

Where the condition of a patient is deteriorating: Escalate the management of these patients is in accordance with the ‘Recognising & Responding to Clinical Deterioration Standard’ and associated practice guidelines.

6. Roles, Responsibilities & Behaviour


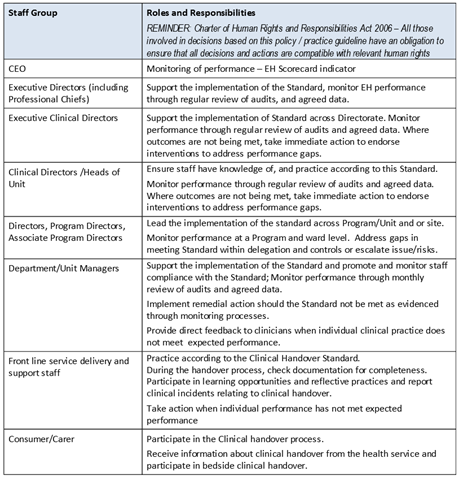


7. Skills, Knowledge & Competencies


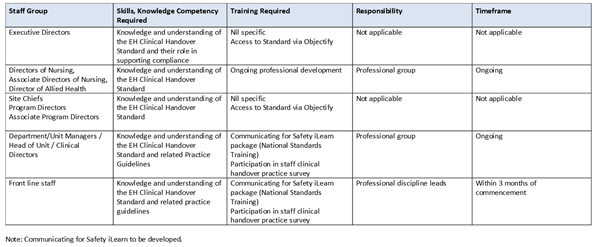


8. Tools & Techniques

ISOBAR Clinical Handover Prompt sheet (Attachment 1)

Electronic Medical Record

Patient Journey Board (Patient Flow Manager, ISOBAR handover sheets)

Eastern Health Intra or Inter-Hospital Clinical Handover / transfer (EH 401510)

Non Eastern Health (external) clinical handover /transfer (EH 275850)

Eastern Health Accreditation intranet portal

OSSIE Guide to Clinical Handover

9. Compliance requirement

National Safety and Quality Health Service Standards, ACSQHC, Standard 6: Communicating for Safety

10. References

• Alfred Health, Clinical Handover Guideline and Clinical Handover Policy

• Austin Health Clinical Handover Policy and Clinical Handover Procedure

• Alvarado, K., Lee, R., Christoffersen, E., Fram, N., Boblin, S., Pool, N., Lucas, J., & Forsyth, S. (2006). Transfer of Accountability: Transforming shift handover to enhance patient safety. Healthcare Quarterly. vol. 9, pp. 75 –79.

• Anderson, C.,D, & Mangino, R.,R. (2006). Nurse Shift Report - Who says you can’t talk in front of the patient? Nursing Administration, vol. 30, pp. 112-122.

• Australian Commission on Safety and Quality in Health Care. National Safety and Quality Health Service Standards guide for hospitals. Sydney: ACSQHC; 2017

• Australian Commission on Safety and Quality in Health Care (ACSQHC) 2011, National Safety and Quality Health Service Standards, ACSQHC, Sydney

• Australian Commission on Safety and Quality in Healthcare (2011). Implementation Toolkit for Clinical handover Improvement, Sydney, ACSQHC <http://www.safetyandquality.gov.au/wp-content/uploads/2012/02/ImplementationToolkitforClinicalHandoverImprovement.pdf>

• Australian Commission on Safety and Quality in Healthcare (2010). OSSIE Guide to Clinical Handover Improvement. ACSQHC, Sydney.

• Chaboyer, W., McMurray, A., Wallis, M. & Chang, H.Y. (2008) Standard Operating Protocol for

Implementing Bedside Handover in Nursing, Griffith University, Australia <http://www.safetyandquality.gov.au/wp-content/uploads/2012/02/SOP-Bedside-Handover.pdf>

• Aldrich R, Duggan A, Lane K, Nair K & Hill, KN (2009). ISBAR revisited: identifying and solving barriers to effective clinical handover in inter-hospital transfer – public report on pilot study. Newcastle: Hunter New England <http://www.safetyandquality.gov.au/wp-content/uploads/2012/01/ISBAR-PSPR.pdf>

• Yee et al “HAND ME AN ISOBAR”: a pilot study of an evidence-based approach to improving shift-to-shift clinical handover. MJA Volume 190 Number 11 1 June 2009 <https://www.mja.com.au/journal/2009/190/11/hand-me-isobar-pilot-study-evidence-based-approach-improving-shift-shift?0=ip_login_no_cache%3D656450823cd63804ee100ba551b458a1>

11. Development History

Developed March 2012 Revised and approved May 2013 into new template. Work of the EAC Clinical Handover – stakeholder representation across the health disciplines and service areas of EH.

Published on objectify 17 Jul 2013 and revised 2015.

Revision in 2018

12. Attachments

Attachment 1: ISOBAR Clinical Handover Prompt Sheet

Attachment 2: Clinical Handover Matrix

**Attachment 1 to the Clinical Handover Standard**

The **ISOBAR** Tool is the approved EH framework for clinical handover communication.

**ISOBAR** provides a flexible standardised framework for clinical handover and can be adapted to reflect the requirements of the specific department or context. ISOBAR can be used in a variety of clinical handover situations. The table below includes a minimum data set for clinical handover and **examples** for bedside handover and phone communication using the ISOBAR framework.

| **I** | **Introduction / Identify**  Patient identification and handover participant identification  *For example*  *Bedside:* Identify the patient (3 Point ID check), introduce self and the team  *Phone:* Introduce yourself, your role in the patient’s care and your reason for calling |
| --- | --- |
| **S** | **Situation**  Current clinical situation (i.e. stable, deteriorating, improving), patient goals or advance care directives, estimated discharge date / discharge destination  *For example*  *Bedside: What brought the patient to hospital; concerns from patient/ family*  *Phone: What is the current situation – If urgent say so, concerns* |
| **O** | **Observations**  This provides an opportunity to identify deteriorating patients  *For example*  *Bedside: recent observations (vital signs, neurological observations, other observations)*  *Phone: recent observations (vital signs, neurological observations, other observations)* |
| **B** | **Background**  Summary of patient background. May include history, presenting problem, background problem, evaluation (investigations, findings, current diagnosis), current issues, management to date, and whether this is working  *For example*  *Bedside: Brief history, date of admission, reason for admission, estimated date of discharge, safety precautions. Allergies*  *Phone: What is the relevant background (pertinent information which may include: Date of admission/Reason for admission/ presenting symptoms or working diagnosis/medications* |
| **A** | **Assessment**  Relevant clinical assessments, any concerns from assessments or outstanding assessments/tasks/actions  *For example*  *Bedside: Clinical assessment / Food and Fluid intake, Urine and Bowel output, Mental Status and Brain function / ADLs, Safety checks/ Clinical Risks (falls, pressure injury, delirium, malnutrition)/Clinical Alerts (aggression, harm to others, infection control), Outstanding assessments, tasks, actions*  *Phone: What do you think the problem is? Clinical assessment, clinical risks, clinical alerts. Outstanding assessments, tasks, actions* |
| **R** | **Request / Recommendations**  Actions required, including responsibility for actions. Recommendations for ongoing care. Agreed transfer of responsibility and accountability for care. Review and clarify information.  *For example*  *Bedside: Review treatment and care plan, review clinical risks, management and recommendations*  *Phone: Be clear about what you are requesting/recommending (phone advice or attendance to the patient or phone handover). Who will be accountable for the care and in what timeframe?*  *Response to referral or consultation is clearly documented in the medical record*  *Any investigations planned and reason for these* |

**Attachment 2 to the Clinical Handover Standard**


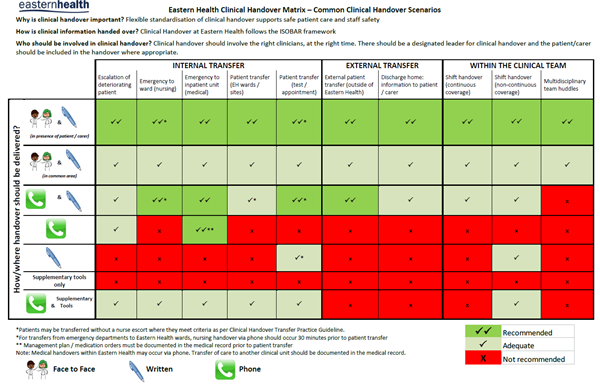

Supplement: Multimedia Appendix 1 [file nursing-v9-e85909-s001.docx]
